# Protocol-developing meta-ethnography reporting guidelines (eMERGe)

**DOI:** 10.1186/s12874-015-0068-0

**Published:** 2015-11-25

**Authors:** E.F. France, N. Ring, J. Noyes, M. Maxwell, R. Jepson, E. Duncan, R. Turley, D. Jones, I. Uny

**Affiliations:** Nursing Midwifery and Allied Health Professions Research Unit, University of Stirling, Unit 13 Scion House, Stirling University Innovation Park, Stirling, FK9 4NF Scotland, UK; School of Health Sciences, University of Stirling, Stirling, FK9 4LA Scotland UK; School of Social Sciences, Bangor University, Bangor, Gwynedd LL57 2DG UK; Nursing Midwifery and Allied Health Professions Research Unit, University of Stirling and Glasgow Caledonian University, Unit 13 Scion House, Stirling University Innovation Park, Stirling, FK9 4NF Scotland UK; Scottish Collaboration for Public Health Research and Policy (SCPHRP), University of Edinburgh, 20 West Richmond Street, Edinburgh, EH8 9DX Scotland UK; Development and Evaluation of Complex Interventions for Public Health Improvement (DECIPHer), Cardiff School of Social Sciences, Cardiff University, 1-3 Museum Place, Cardiff, CF10 3BD UK

**Keywords:** Reporting guideline, Meta-ethnography, Qualitative review or synthesis, Qualitative research, Evidence-based practice, Systematic review

## Abstract

**Background:**

Designing and implementing high-quality health care services and interventions requires robustly synthesised evidence. Syntheses of qualitative research studies can provide evidence of patients’ experiences of health conditions; intervention feasibility, appropriateness and acceptability to patients; and advance understanding of health care issues. The unique, interpretive, theory-based meta-ethnography synthesis approach is suited to conveying patients’ views and developing theory to inform service design and delivery. However, meta-ethnography reporting is often poor quality, which discourages trust in, and use of, meta-ethnography findings. Users of evidence syntheses require reports that clearly articulate analytical processes and findings. Tailored research reporting guidelines can raise reporting standards but none exists for meta-ethnography. This study aims to create an evidence-based meta-ethnography reporting guideline articulating the methodological standards and depth of reporting required to improve reporting quality.

**Methods/design:**

The mixed-methods design of this National Institute of Health Research-funded study (http://www.stir.ac.uk/emerge/) follows good practice in research reporting guideline development comprising: (1) a methodological systematic review (PROSPERO registration: CRD42015024709) to identify recommendations and guidance in conducting/reporting meta-ethnography; (2) a review and audit of published meta-ethnographies to identify good practice principles and develop standards in conduct/reporting; (3) an online workshop and Delphi studies to agree guideline content with 45 international qualitative synthesis experts and 45 other stakeholders including patients; (4) development and wide dissemination of the guideline and its accompanying detailed explanatory document, a report template for National Institute of Health Research commissioned meta-ethnographies, and training materials on guideline use.

**Discussion:**

Meta-ethnography, devised in the field of education, is now used widely in other disciplines. Methodological advances relevant to meta-ethnography conduct exist. The extent of discipline-specific adaptations of meta-ethnography and the fit of any adaptions with the underpinning philosophy of meta-ethnography require investigation. Well-reported meta-ethnography findings could inform clinical decision-making. A bespoke meta-ethnography reporting guideline is needed to improve reporting quality, but to be effective potential users must know it exists, trust it and use it. Therefore, a rigorous study has been designed to develop and promote a guideline. By raising reporting quality, the guideline will maximise the likelihood that high-quality meta-ethnographies will contribute robust evidence to improve health care and patient outcomes.

## Background

Department of Health policy [1] in the United Kingdom (UK) states that evidence-based decision making requires both qualitative *and* quantitative research. Because of the large amount of research evidence available, both types of evidence need to be robustly synthesised [1] in ways that can be used for designing and implementing high-quality, health care services, interventions and programmes to improve patient care. The National Institute for Health and Care Excellence (NICE) guideline for patient experience in the National Health Service (NHS) [2] and UK government White Paper ‘Equity and excellence: liberating the NHS*’* [3] stress that improving patients’ experience of care is key. Syntheses of quantitative studies can provide evidence of the epidemiology of disease and conditions as well as intervention effectiveness. Qualitative evidence syntheses, such as meta-ethnography, can provide evidence of what it is like to experience a disease or condition; feasibility, appropriateness and acceptability to patients of interventions or services [4, 5]; and inform implementation of complex interventions (e.g., by explaining why interventions effective in trial settings are not implemented in mainstream practice). Syntheses of qualitative studies (which we refer to in this article as ‘qualitative synthesis’ or ‘qualitative evidence synthesis’) can also illuminate complex health care issues by developing theory about how a health service, policy, strategy or intervention works or not and how patients experience it [6] and advance understanding of patients’ experiences of any complex healthcare issue [6] or illness [7–10], for example, what it is like to have and be treated for arthritis.

Currently, individual qualitative studies often rank low in the clinical evidence hierarchy [11] because the focus of such hierarchies is on the level of bias which could affect effectiveness estimates. Whilst qualitative studies can never be used to assess effectiveness, they are essential for understanding patient experiences; rigorously synthesising them can increase their importance and relevance in the evidence base, and thus help to ensure their contribution to informing clinical guidelines [12], service design and care.

### What is meta-ethnography?

Meta-ethnography [13] is a unique, interpretive, qualitative synthesis approach suited to conveying patients’ views and experiences and informing implementation of services and interventions. It was developed by Noblit and Hare [13], in the field of education, in the 1980s. Most qualitative synthesis approaches are based on meta-ethnography [14], which is the most widely used approach in health-related research [15]. A recent evaluation concluded that, if well-conducted and reported, meta-ethnography is effective, systematic and can produce important new conceptual understandings of complex healthcare issues, even in well-researched fields [16]. Thus, meta-ethnography has great potential to improve health services, public health, and understanding of patient experiences [7–10] for any health condition or service.

In a meta-ethnography, the reviewers conducting the meta-ethnography aim to produce new interpretations that transcend the findings of individual studies, rather than simply to aggregate findings. Reviewers systematically compare study concepts to identify new overarching concepts. The approach allows reviewers to preserve the original meanings of study concepts and take account of contextual impacts on findings [13, 16]. Meta-ethnography differs from other qualitative evidence synthesis approaches in its underpinning theory [17], use of the authors’ interpretations (e.g. concepts, themes) from primary qualitative studies as data, and creation of new interpretations through its unique, systematic analytic synthesis process.

The meta-ethnography approach continues to evolve. The originators’ [13] synthesis process was somewhat unclear [7] but detailed worked examples of meta-ethnographies on health-related topics were published in the early 2000s [9, 18]. The originators also gave no guidance on how to sample or appraise studies for inclusion, although robust methods to identify [19] and select studies now exist [20] which can be applied to the conduct of meta-ethnography as well as to syntheses of all kinds. Some recent guidance has also been developed on if and how to conduct quality appraisal of studies for inclusion in a meta-ethnography [21] and on how to select a qualitative synthesis approach to suit a research aim [22–24]. However, consensus on good practice in meta-ethnography conduct and reporting is lacking.

### Quality of meta-ethnography reporting

**Fig. 1:**
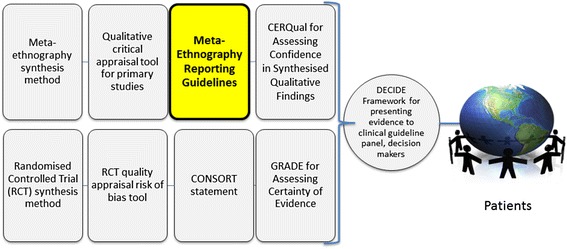
Role of meta-ethnography reporting guidelines in facilitating use of synthesised research evidence

Users of evidence syntheses, such as patient groups, health service managers, policy makers and clinicians, require quality reports that clearly articulate the analytical processes and findings, but the reporting quality (rigour and transparency) of meta-ethnographies varies and is often poor [15, 18], reducing the potential utility of such research [25]. Systematic reviews assessing meta-ethnography reporting quality [15, 18] show this is not improving: over two-thirds of recent, peer-reviewed, health-related meta-ethnography journal articles did not clearly describe their analysis and synthesis processes, i.e. how they analysed concepts from primary studies [15, 18]. Quality reporting is a prerequisite to assessing confidence in [19], and so trusting and enabling use of meta-ethnography findings to enhance health care and services (see Fig. 1).

### The need for a reporting guideline for meta-ethnography

There is no bespoke reporting guideline for meta-ethnography despite its unique, complex analysis methods and dominance as a qualitative synthesis approach in health-related research [15, 25]. A generic reporting guideline for qualitative syntheses exists - the 2012 ENTREQ statement [26] for enhancing transparency in qualitative evidence synthesis reporting - but it has limitations: it was not developed using consensus methods with qualitative synthesis experts and was not designed for the conceptually, methodologically distinct meta-ethnography approach with its unique synthesis process. ENTREQ is not being used widely to inform, and is unlikely to greatly improve, meta-ethnography reporting because it provides no guidance on how to report the analytic synthesis process (and so cannot simply be adapted for meta-ethnography) [18]. Only one of 32 recent meta-ethnography journal articles used ENTREQ to guide reporting and its analytic reporting was unclear [18]. Tailored research reporting guidelines, such as CONSORT [27] for randomised controlled trials and PRISMA for systematic reviews and meta-analyses [28], are widely used and can raise reporting standards [29]. Because qualitative evidence synthesis approaches differ greatly, the need for specific guidelines for reporting other unique forms of qualitative evidence synthesis (realist and meta-narrative reviews) has been recognised and these have recently been developed [30, 31].

## Methods/design

### Aim

The aim of this study is to create an evidence-based meta-ethnography reporting guideline that articulates the methodological standards and depth of reporting required to improve reporting quality and transparency. The purpose of the guideline is to maximise the value and utility of meta-ethnographies for enhancing health service design and delivery, so improving patient experiences and outcomes for any specific health service, issue or illness.

### Research questions

1. What are the existing recommendations and guidance for conducting and reporting each process in a meta-ethnography, and why?
2. What good practice principles can we identify in meta-ethnography conduct and reporting to inform recommendations and guidance?
3. From the good practice principles, what standards can we develop in meta-ethnography conduct and reporting to inform recommendations and guidance?
4. What is the consensus of experts and other stakeholders on key standards and domains for reporting meta-ethnography in an abstract and main report/publication?

### Study design/ methods

**Fig. 2:**
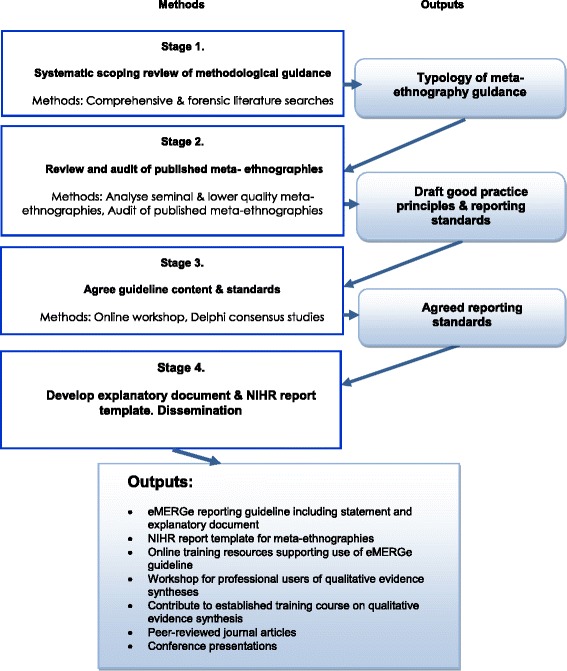
eMERGe mixed methods research design

Our mixed-methods study design follows key steps recommended as good practice in health-related research reporting guideline development [32] that have been used successfully to develop reporting guidelines for other qualitative evidence synthesis approaches (realist and meta-narrative reviews) [30, 33]. The key steps include: literature reviews of relevant guidance (Stage 1) and of actual reporting practice in published research articles (Stage 2), a guideline item development workshop and Delphi consensus studies with experts (Stage 3), developing a guidance statement and accompanying explanatory document (Stages 1–4), and encouraging guideline endorsement by journals (Stage 4). The project will involve four main stages, illustrated in Fig. 2, which are now described in detail.

#### Stage 1. Identifying recommendations and guidance

**Fig. 3:**
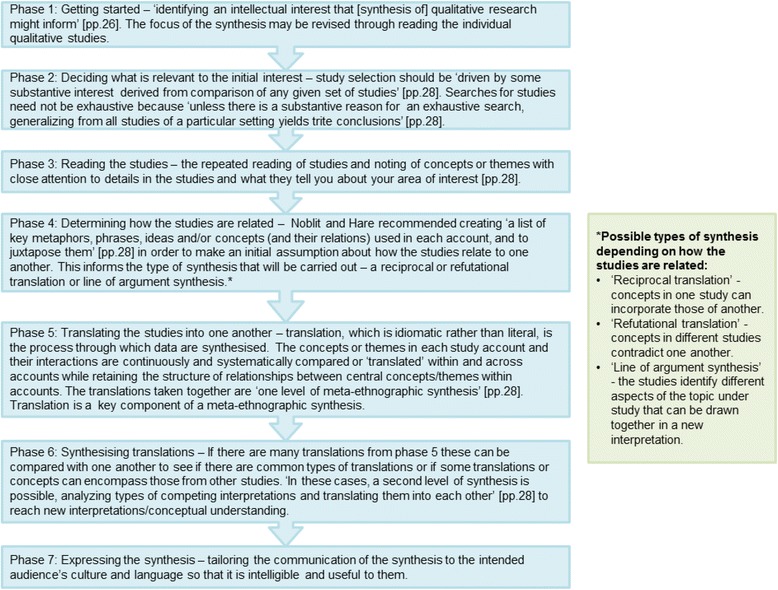
The seven phases of Noblit and Hare’s [13] meta-ethnography approach

A methodological systematic review of the literature, including ‘grey’ literature such as reports, doctoral theses and book chapters, will be conducted to identify existing guidance and recommended practice in conducting and reporting meta-ethnography from any discipline. This review has been registered on PROSPERO, the International Prospective Register of Systematic Reviews, (registration number: CRD42015024709). The review is the first step in identifying what should be included in the reporting guideline and will result in a preliminary definition of what constitutes a meta-ethnography and an initial detailed typology of recommendations and guidance regarding the elements in a meta-ethnography that could be reported. Our key focus will be on the meta-ethnography analytic synthesis phases 4 to 6 (Fig. 3 gives a description of all the seven phases in a meta-ethnography), which are complex and currently very poorly reported [15, 25]. This stage is designed to answer research question 1.

#### Search strategy

An exhaustive search strategy will combine comprehensive database searches with forensic or ‘expansive’ searches. These searches will be iterative and will evolve as the review progresses because their purpose is to build our knowledge of recommendations and guidance in conducting and reporting meta-ethnography rather than to answer a tightly-defined research question [20]. This will combine browsing of texts (publications) with periods of more focused systematic searching rather than a linear search process [20].

To identify relevant literature we will start with seminal methodological and technical publications known to our international expert academic advisors and the project team including Noblit and Hare’s [13] seminal original book on meta-ethnography and detailed worked examples of meta-ethnographies. Relevant texts from a range of disciplines that use meta-ethnography including health, education and social work will be included. Citation searching, reference list checking (also known as backward and forward ‘chaining’) of the seminal texts; searches of key websites, such as the Cochrane library; searches of Google Scholar; and searches by names of authors of relevant publications will be performed. Comprehensive database searches to identify other methodological publications will also be conducted.

#### Comprehensive database searches to identify methodological publications

Bibliographic databases will be searched including: MEDLINE, SCOPUS, PsycARTICLES, PsycINFO via Health Source: Nursing/Academic Edition; Pubmed; CINAHL; the International Bibliography of the Social Sciences; Sociological abstracts; Web of Science Core Collection; British Education Index; ERIC (Educational Resources Information Center); Australian Education Index; and ERA (Educational research abstracts) Online. The draft search terms are shown in Table 1 – these will be refined following piloting. The final search strategy will be available from the corresponding author after completion of the project. Reviewers will also hand search reference lists in texts that meet the inclusion criteria for the review for other relevant studies not already identified.

**Table 1:** Draft search strategy for Medline for Stage 1 methodological systematic review

1	metaethnograph*.mp.
2	meta ethnograph*.mp.
3	Meta-ethnograph*.mp.
4	qualitative evidence synthes?s.mp.
5	noblit.mp.
6	(qualitative adj2 (review or systematic or overview)).mp.
7	(“third order” adj2 construct*).mp.
8	(“line* of argument” or “line*-of-argument”)
9	(metanarrative or meta narrative or meta-narrative or metasynthes?s or meta synthes?s or meta-synthes?s).mp.
10	or/1–9
11	((good or best or recommend* or quality) adj3 (guid* or design or standards or practice or practices or reporting or method*)).mp.
12	((publishing or reporting) adj2 (guid* or design or standards or practice or practices or method*)).mp.
13	Publishing/st [Standards]
14	methods/st
15	11 or 12 or 13 or 14
16	10 and 15

When searching bibliogrpahic databases 'wildcards' are used in a search query to represent unknown characters.In MEDLINE, the asterisk (*) represents any group of characters, including no character and a question mark (?) represents any single character, e.g. 'meta synthes?s' will find 'meta syntheses' and 'meta synthesis.'

#### Screening and selection of texts

One reviewer will perform initial screening by title to exclude off-topic texts, i.e. those clearly not about meta-ethnography or evidence synthesis. Two reviewers will independently screen potentially relevant texts first by title and abstract and, if necessary, by full text using the inclusion and exclusion criteria. Disagreements over inclusion/exclusion will be resolved through discussion. A third reviewer will also screen texts if the first two reviewers cannot reach agreement. The inclusion criteria are:

- A book, book chapter, journal article, editorial, report, or doctoral thesis
- That reports on methodological issues in conducting meta-ethnography OR is a reporting guideline for or provides guidance on reporting qualitative syntheses including meta-ethnography
- Any topic or focus, not just health-related (e.g. education, social work)
- Published after 1988 when Noblit and Hare’s [13] book came out.
- In any language.

#### Data extraction and analysis

Data will be extracted from each included text by only one reviewer because this is a qualitative review in which the key principles are transparency and consensus, not independence and inter-rater reliability. The completeness of the data extraction will be checked by a second reviewer for five texts per reviewer (10 in total). A template using Microsoft Excel will be developed and piloted to record characteristics of publications including details such as the authors, publication year, title, aim, topic focus, academic discipline, and type of publication. The initial data extraction categories will be informed by the project team’s earlier methodological review [18] and will be refined following piloting.

Extracted data will be analysed qualitatively. The qualitative analysis software NVivo 10.0 [34] will be used to facilitate management and coding of documents. Data will be read repeatedly, annotated, and we will write and iteratively revise descriptive memos (analytical notes) to articulate ideas and arguments. We will analyse: how meta-ethnography is defined and differs from other qualitative evidence synthesis approaches; how to identify that meta-ethnography is the appropriate method for a particular research question; the range of practices, recommendations and guidance on how to do the seven meta-ethnographic phases (described in Fig. 3), especially the analytic phases 4 to 6 and their component tasks; how to conduct a meta-ethnography and present findings so that they are useful/relevant for health and social care policy, practice and decision making; definitions of reciprocal and refutational analysis and line of argument synthesis; how to develop theory after the analytic synthesis; how to handle the unique context of primary studies in analysis and synthesis; and authors’ views, if any, on good and bad practices. Any additional steps in meta-ethnography conduct identified (i.e. in addition to Noblit and Hare’s seven phases) will be recorded and any areas of consensus, divergence, ambiguity or lack of evidence among texts (publications) identified.

The guidance identified will inevitably vary in quality and the detail it provides, but there is currently no tool for appraising the quality of meta-ethnographies to establish methodological rigor. Therefore the full range of practice will be documented, regardless of the richness (quality) of the text, but we will record whether texts are rich in detail, i.e. a detailed account with in-depth explanation and rationales that goes beyond description.

From Stage 1 a narrative synthesis will be produced containing a preliminary definition of what constitutes a meta-ethnography and a detailed typology of recommendations and guidance for meta-ethnography conduct and reporting. This will be shared with our project advisory group of academic experts and other stakeholders, such as professional users of meta-ethnographies and lay people, who will be asked for their feedback to enhance the external validity of the findings. Recommendations and guidance may or may not represent good practice, therefore meta-ethnographies considered by experts to be seminal or of lower quality will be examined in Stage 2 to identify good practice principles and develop standards.

#### Stage 2. Defining good practice principles and standards

In Stage 2 the existing guidance identified in Stage 1 will be compared to actual practice in published meta-ethnographies in order to identify and develop good practice principles and standards in their conduct and reporting. This will form the basis of provisional reporting guideline standards and items for the expert and stakeholder panels to consider in Stage 3. Stage 2 will entail:

- Stage 2.1 – a review and analysis of seminal and lower quality (in conduct and/or reporting) meta-ethnographies and
- Stage 2.2 – an audit of published, peer-reviewed meta-ethnography journal articles.

**Fig. 4:**
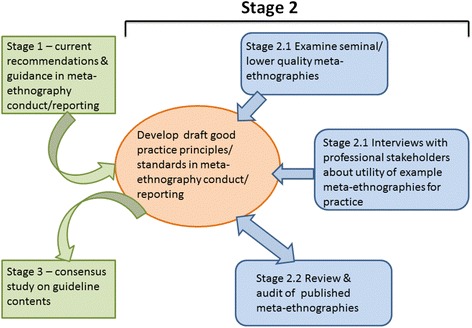
Components of Stage 2 - drafting good practice principles and standards in meta-ethnography conduct and reporting

Stages 2.1 and 2.2 will now be described in detail. Figure 4 shows the different components of Stage 2 and how they relate to each other and to Stages 1 and 3. The aims of Stage 2 are to identify good practice principles and to develop standards in meta-ethnography conduct and reporting to inform recommendations and guidance. This stage is designed to answer research questions 2 and 3.

#### Stage 2.1 Review of seminal and lower quality meta-ethnographies

In order to identify examples of good practice, our expert academic advisors will be asked to recommend published, peer-reviewed meta-ethnography journal articles from any discipline. The articles must have been published following Noblit and Hare’s book in 1988 and be ones that they consider to be seminal, i.e. meta-ethnographies that have influenced or significantly advanced thinking and/or that are of central importance in the field of meta-ethnography, and advisors will be asked to explain why they chose the articles. The experts will also be asked to identify any published, peer-reviewed meta-ethnography journal articles that they consider to be relatively poorly conducted and/or reported and to explain why, as a comparison. A list of seminal and lower quality meta-ethnographies will be collated - if necessary, advisors will agree a final list of the 10 to 15 ‘best’ or ‘worst’ examples of each. Overall, 10 to 15 seminal and 10 to 15 lower quality (in conduct and/or reporting) meta-ethnographies will be reviewed.

The utility of the meta-ethnographies for key stakeholders who are potential professional end users of meta-ethnography evidence syntheses - such as clinical guideline developers and staff from organisations that produce clinical guidelines or use health research evidence - whose needs may differ compared to those using it for academic purposes, will be investigated to identify elements of reporting that are important to them. Ten professional end users will be invited to comment on the utility for practice and policy of the identified seminal and lower quality meta-ethnographies. Each stakeholder will be sent a copy of one seminal meta-ethnography and one lower quality meta-ethnography (selected for likely relevance to the individual). They will be asked for qualitative feedback relating to how useful they found the findings and the way in which the meta-ethnography was reported and how reporting could have been improved. Data will be collected via email or telephone, depending on the participant’s preference, in a semi-structured interview format.

#### Stage 2.1 data analysis

Data analysis of the seminal and lower quality meta-ethnographies will focus on what and how the authors of each meta-ethnography conducted and reported each phase of a meta-ethnography, especially the analysis phases 4 to 6. We will identify the range of approaches, compare and contrast their approaches with recommendations and guidance identified from the methodological texts in Stage 1, identify any examples of how seminal texts have advanced the meta-ethnography method, and define good and poor reporting practices. Characteristics of the meta-ethnographies will be recorded including the authors, title, journal details including article word limits, publication year, the main topic focus, aim of the review, and the number of studies synthesised. For rigour and to enhance richness of interpretation, four members of the project team with meta-ethnography expertise will contribute to data analysis. The content of telephone interviews with professional end users will be documented via detailed note-taking and the issues collated to identify what represents good reporting to them.

Included meta-ethnography texts will be qualitatively analysed deductively and inductively. The initial deductive coding frame of analytic categories will be based on the typology identified in Stage 1 and will be refined as new codes are developed inductively from the data and from interviews with professional end users. The suitability of the approach for the study aim, the rationale for the use of meta-ethnography, how each phase in the meta-ethnography was conducted and reported, and good practice in any area of doing or reporting a meta-ethnography are topics likely to be analysed.

The analysis of the usefulness of and the actual practice in the seminal and lower quality meta-ethnographies will be juxtaposed with the recommendations and guidance from Stage 1 to identify commonalities and differences. This will allow us to identify good practice principles and to develop standards against which to compare recent meta-ethnographies in Stage 2.2. An example of a possible standard that might be produced is that meta-ethnography publications should include a clear justification of why the reviewers selected the meta-ethnography approach to address their research aim.

#### Stage 2.2 Audit of published meta-ethnographies

The literature will be audited to examine how published meta-ethnographies perform against the standards developed from the good practice principles identified in stage 2.1 and to further develop these principles and standards. Database searches will be conducted to identify published, peer-reviewed, health-related or social care meta-ethnography journal articles published since 1994. No peer-reviewed, health-related meta-ethnography journal articles were published before 1994 (10). The aim is to identify a broad range of meta-ethnographies from which to purposively sample articles. Electronic databases will be searched including: MEDLINE, PsycARTICLES, PsycINFO via Health Source: Nursing/Academic Edition; Pubmed; the International Bibliography of the Social Sciences; Sociological abstracts; Applied Social Sciences Index and Abstracts; and CINAHL. Draft search terms are shown in Table 2 – these will be refined following further piloting. The final search terms can be requested from the corresponding author after conduct of the project.

**Table 2:** Draft search strategy for Medline for Stage 2 systematic literature review and audit

1	metaethnograph*.mp.
2	meta ethnograph*.mp.
3	Meta-ethnograph*.mp.
4	qualitative evidence synthes?s.mp.
5	noblit.mp.
6	(qualitative adj2 (review or systematic or overview)).mp.
7	(“third order” adj2 construct*).mp.
8	(“line* of argument” or “line*-of-argument”)
9	(metanarrative or meta narrative or meta-narrative or metasynthes?s or meta synthes?s or meta-synthes?s).mp.
10	or/1–9

When searching bibliogrpahic databases 'wildcards' are used in a search query to represent unknown characters.In MEDLINE, the asterisk (*) represents any group of characters, including no character and a question mark (?) represents any single character, e.g. 'meta synthes?s' will find 'meta syntheses' and 'meta synthesis.'

#### Stage 2.2 screening and sampling of articles

Two independent reviewers will screen articles by title and abstract and, if necessary, by full text. If they cannot reach agreement, a third reviewer will also screen the text. To be relevant to the review, an article must meet all of the following criteria:

- In the title, abstract and/or main manuscript have described their methods as meta-ethnography or as using the methods of Noblit and Hare
- Be reporting a synthesis of qualitative primary research studies
- Focusing on a topic with a health, health care or social care focus
- Published in a peer-reviewed journal
- Published after 1994.

Around 200 to 250 relevant meta-ethnographies are likely to be retrieved. From these a diverse sample of 40 meta-ethnographies will be purposively selected. The purpose is knowledge building therefore purposive sampling is appropriate. The sample of 40 meta-ethnographies will include articles from a range of different journals, with a variety of main focuses (e.g. experiences of a health condition or health service, health professionals’ experiences, health promotion, public health, social care); from a variety of different disciplinary backgrounds (e.g. nursing, midwifery, sociology, psychology, allied health professions, social work); and a range of publication dates after 1994. At least two articles will be selected because they are based on longer reports, e.g. reports to funders, to allow us to compare the methodological reporting in the article versus the report to get insight into the limits imposed by journal formats and word limits.

#### Stage 2.2 data analysis

Characteristics of the meta-ethnographies, e.g., title and authors, will be recorded in the same bespoke template that will be developed and used in Stage 2.1. Data will be extracted from included texts into a coding frame based on the draft standards and will be analysed qualitatively to judge if they meet the standards identified in Stage 2.1. The coding frame will be revised to allow for refinement of and additions to the principles and standards as new codes are developed inductively from the data.

A detailed analysis will be produced comparing the conduct and reporting in recent meta-ethnographies to the standards to see whether and how they follow good practice, to identify any additional good practices, and identify examples of how the method has been further advanced, for example, in how the analysis processes are reported. Analysis will also identify barriers to quality reporting - such as abstract and manuscript word limit, journal reporting templates, and how authors apportioned word limit - and common pitfalls, for instance, inappropriate application of meta-ethnography. The findings will be used to refine the descriptions of recommendations, guidance and good practice principles and standards. These will be used as the basis for drafting a list of potential reporting items and standards relevant to the conduct and reporting of a meta-ethnography which the expert and stakeholder panels will consider and rate in Stage 3.

#### Stage 3. Agreeing guideline content

Recommended good practice in developing research reporting guidelines involves expert input and the use of expert consensus in agreeing their contents [32]. This is the approach adopted in this study. For further rigour and to ensure a guideline useful to health services and patients, we will include an extra element which other reporting guideline developers have not done, which is to include in the consensus process an equal proportion of other key stakeholders/potential users of meta-ethnographies including the public and patients, policy makers, clinical guideline developers, commissioners of meta-ethnographies, and clinicians. This stage is designed to answer research question 4.

#### Stage 3 has two parts

- Stage 3.1. An online expert and stakeholder workshop to further develop and generate potential items for inclusion in the reporting guideline that will be subsequently rated in online Delphi (‘eDelphi’) studies.
- Stage 3.2. Two eDelphi studies to reach consensus on the items (generated in Stage 3.1) to be included in the reporting guidelines.

#### Stages 3.1 & 3.2. Sampling and Sample

Two main groups of participants will be recruited: (a) 45 academic experts and (b) 45 other key stakeholders who use synthesised evidence, i.e. people who use evidence in a professional capacity and patient and public representatives. Previous reporting guidelines have been successfully developed with a total of 30 participants [32].

#### Academic experts

An international, multi-disciplinary panel of 45 methodological experts in qualitative evidence synthesis and meta-ethnography will be purposively recruited via professional networks, inviting authors of key texts and asking experts to suggest participants (‘snowballing’). Sixty-five experts will be invited by email to participate in both stages 3.1 and 3.2. A recruitment rate of 70 % (*N* = 45) and attrition of 33 % are anticipated during stages 3.1 and 3.2 [30, 31] giving a final sample of at least 30 experts.

#### Other key stakeholders

A diverse United Kingdom (UK) sample of 45 key stakeholders will be purposively recruited including 22 to 23 public/patient representatives (aged ≥16) and 22 to 23 other stakeholders (potential professional users of qualitative evidence syntheses) to take part in the panel. Members of the public and patients and their representatives will be identified and invited through voluntary and patient organisations. Professional evidence users (e.g. clinical guideline developers, policy makers) will be identified through relevant organisations. By email, telephone and in person, 60 professionals will be approached to recruit 22 to 23 (anticipated recruitment rate of around 40 %). Attrition of 33 % during Stage 3 is anticipated resulting in a final sample of at least 30 key stakeholders (in addition to the 30 academic participants). For all types of stakeholders we will also draw on our existing contacts and networks, our advisory group’s networks, ‘snowballing’ and on adverts to recruit participants.

#### Stage 3.1. Online workshop

In an online workshop lasting three to four hours, the project team will interact with the panels of experts and other stakeholders to discuss good and best practice, issues of controversy or ambiguity in meta-ethnography conduct and reporting, and to further develop the draft standards and items for the reporting guideline and agree their wording. An online format has been selected for inclusivity of participants, efficiency and economy.

#### Procedure

This workshop will underpin the reporting guideline development and ensure that the panels have up-to-date knowledge about meta-ethnography and the quality of its reporting. This is an online equivalent to the face-to-face expert meeting recommended for reporting guideline development [32]. We anticipate that only 40 to 50 % of the 90 panel members will be available to participate on the day. If necessary, we will run two similar workshops in order to accommodate panel members based in different time zones. The workshop will be chaired by an experienced group facilitator. From the findings of Stages 1 and 2 the project team will present relevant background information on qualitative evidence synthesis and the characteristics of meta-ethnography; a summary of evidence on meta-ethnography guidance, practice and reporting; and suggest specific reporting guideline standards and items that are being proposed for rating in the eDelphi studies along with relevant empirical evidence and justification.

#### Data collection and analysis

Presentations will be followed by open debate, questions, brainstorm, exchanges of views and knowledge, and discussion [32]. The definition of a meta-ethnography, how close standards and items are to best practice, and whether further improvement is needed will be explored. Comments will be solicited from other stakeholders on the utility of meta-ethnography reports for improving clinical practice and intervention implementation. Participants will have the opportunity to suggest further guideline standards and items for inclusion in the eDelphi studies, identify and combine duplicative standards/items, and revise the wording of items.

Panel members’ feedback on the proposed standards and items for the guideline will be systematically recorded and thematically analysed, noting if they are an academic expert or other stakeholder. A summary of issues, topics and themes will be created in a matrix structured according to the seven key phases in meta-ethnography conduct. A list of standards and items for potential inclusion in the reporting guideline will be compiled to be taken forward to the eDelphi studies. This list will be circulated to the panels prior to the start of the eDelphi to agree and finalise wording of items.

#### Stage 3.2. Online Delphi studies

Delphi is a group consensus-reaching method, originally developed by the RAND Corporation in the 1950s [34], which presents questionnaires in a series of rounds, each one based on feedback from respondents’ responses to the previous version of the questionnaire [35]. The Delphi study method has been used extensively in health care research and in guideline development [36–38]. A key advantage of the Delphi method is that the anonymity of participants’ responses avoids peer-group pressure to conform to the majority view so it encourages honest, unbiased opinions. Furthermore it does not require face-to-face interaction which would be challenging and costly with a geographically-spread panel. The aim of these Delphi studies is to achieve expert and/or key stakeholder consensus on the content of the reporting guideline. The studies will determine which items are the most important to include in a reporting guideline covering the abstract and main manuscript.

#### Procedure

The sample and sampling have been described already. We anticipate that 30 out of 45 participants (60 out of 90 overall) will complete all rounds in each eDelphi, a sample comparable to published Delphi studies. Two separate but related eDelphi studies will be run in parallel - one for academic experts and one for other stakeholders. Carrying out two separate studies will ensure that the views of both groups can be differentiated between and represented, so that items of importance to both groups will be included in the guideline. The two eDelphi studies will involve participants rating the same guideline items and the procedure of the eDelphi studies will be exactly the same for each participant group**.**

We will use a web-based platform (The Stirling eDelphi Platform^**©**^) developed at the University of Stirling specifically for eDelphi studies which has been piloted for acceptability and usability and successfully used in previous eDelphi studies [39, 40]. This web-based platform has automatic reminders, collation, analysis and feedback functions which considerably increase efficiency by reducing the administration and manual analysis that is normally required between Delphi study rounds. Rates of study participation are comparable to paper-based administration methods [39].

**Fig. 5:**
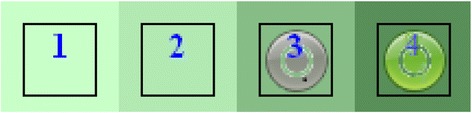
An example of a colour histogram of previous responses from The Stirling eDelphi Platform^©^

Upon logging in to the eDelphi website, the participant is presented with the questionnaire. In rounds 2 and 3 the participant sees the same items they rated in the previous round plus any additional items subsequently suggested by participants. They also see the most popular response (s) to each item from the previous round, their own previous response, and the relative frequency of responses for each item. These data are presented visually in the form of a colour histogram or ‘heat map’ which overcomes some of the known limitations of using measures of central tendency [41] when feeding back results to participants, e.g. when the median score disguises that consensus is polarised. Figure 5 is an example histogram showing the frequency with which each of four responses was chosen in a previous round (the darker the shade of green, the greater the number who selected that response; the lighter the shade of green, the fewer the number). The grey circle shows the choice that the current participant made in the previous round and the green circle shows the choice that they have made in the current round (in round 1 each box is white because no previous selections have been made).

#### Data collection

Data collection will take 12 weeks in total and takes place over three rounds, each lasting four weeks. Having three rounds avoids excessive participant fatigue and maximises the potential to reach consensus amongst participants [41]. Electronic reminders will be sent automatically to participants two weeks after the commencement of each round, and also shortly before the end of the round to individuals who have not yet completed the round. These reminders state the final date by which the current round must be completed.

In each round a set of provisional items (agreed in Stage 3.1) will be presented on the eDelphi website. Each participant will be asked to rate how important they consider it to be (on a four point Likert-type scale from very important to very unimportant) that the item should appear in the guideline. Participants will have the option to state that they have no expertise related to any item listed. In round 2 participants will have the option to add items which they consider important that are not already listed in round 1. No qualitative data will be collected, other than suggestions for additional guideline items.

#### Data analysis

Inter-round data collation is completed automatically and fed back to participants during the subsequent round, as described already. Following completion of round 3 (the final round), descriptive statistics of the ordinal data (frequencies/ percentage of responses) showing the level of consensus for each item for each study will be prepared. There will be two final sets of consensus ratings from the two parallel Delphi studies. Non-parametric inferential statistics (the Wilcoxon Signed Ranks Test) will be used to assess whether there is a statistically significant increase in consensus for item importance between rounds for each study. Items with a final consensus level of ≥70 % (for very important/important response categories) in *either* of the eDelphi studies (i.e. items considered important by a majority of *either* group) will appear in the guideline [40, 41]. An item/standard will only be excluded from the guideline if neither group rated it as important or very important. This means that as long as an item is considered important to include by ≥70 % of participants in at least *one* of the participant groups then it will appear in the guideline. We will produce the final version of the meta-ethnography reporting guideline standards and items (the ‘guideline statement’) which will be fed back to the panels via email.

### Ethical issues

Stages 1 and 2 of the study are deemed exempt from the need for ethical approval (personal communication J Evans 3.3.15, School of Health Sciences Research Ethics Committee, University of Stirling). Ethical approval for Stage 3 of the project has been granted by the School of Health Sciences Research Ethics Committee at the University of Stirling (SREC 14/15 – Paper No 31 – Version 1). In Stage 3 all potential participants will be given a letter of study invitation and a study information sheet. They will have the opportunity to ask questions about the study before deciding whether to take part. Participants will agree at the outset how their intellectual contribution to the study will be recognised in project outputs such as publications.

Participation in the online workshop and eDelphi studies will be taken as participants’ implicit consent. Participants will be informed in the information sheet and verbally at the start of the workshop that it will be audio-recorded and detailed minutes taken, that we might use anonymised verbatim quotations in project publications (unless they prefer to be identified), and that they can choose not to participate if they do not want to be recorded. Participants will be told that they can retract their contributions to the workshop and can withdraw from the study at any time and that this will not affect their work or health care.

It is unlikely that the research topic will cause emotional distress to participants or researchers. There is a slight possibility that participants and researchers with experience of a health issue might become upset if the study causes them to reflect on their experience of any health issues that affect them or people close to them. We will provide participants with a list of potential sources of support and offer them the option to take a break from or discontinue their participation. Researchers will receive regular supervision and debrief sessions.

#### Stage 4. Develop explanatory document, National Institute for Health Research (NIHR) report template, and dissemination

A detailed explanatory document, drawing on findings from Stages 1 to 3, will be produced to accompany the guideline statement. The explanatory document will provide detailed rationales and evidence for all of the guideline items and standards. For each item, the document will include (a) an example of good reporting from a published article, and (b) the scientific background and rationale for including that information in a published article. The document will be published simultaneously along with the guideline statement. The document will be circulated to the expert and stakeholder panels for comments and feedback prior to journal submission.

Based on the contents of the reporting guideline and explanatory document an NIHR report template for commissioned meta-ethnographies will be developed. The current NIHR template for evidence syntheses and systematic reviews is designed for syntheses of quantitative studies, such as systematic reviews of intervention effectiveness. Online training resources supporting use of the guideline will also be created and a workshop about the guideline run for professional users of qualitative evidence syntheses. Other dissemination activities are described in Fig. 2.

## Discussion

Meta-ethnography was originally developed in the 1980s by Noblit and Hare [13] to synthesise ethnographic studies in the field of education. Since it was developed, meta-ethnography has been used in a range of other academic fields, such as health and social care, and applied to the synthesis of a variety of types of qualitative study, not just ethnographies [8, 9]. Applying or ‘translating’ the approach for a specific discipline has typically been undertaken by reviewers whilst conducting a meta-ethnography; undoubtedly there will be a range of practices of varying quality. However, we do not know yet to what extent discipline-specific adaptations have been made or to what extent any approaches commonly used to operationalise meta-ethnographic principles within different academic disciplines accord with the thinking of the originators Noblit and Hare. In order to capture the conceptual and philosophical underpinnings of meta-ethnography and the implications of these for subsequent adaptations, the project team will work closely with Professor George W. Noblit who will join key meetings at strategic points in the study.

Since 1988 when Noblit and Hare published their book on meta-ethnography there have been relevant methodological advances. For instance, Noblit and Hare gave no detail on how to implement the synthesis process [7] and no guidance on how to sample or appraise studies for inclusion; subsequently rigorous methods for identifying and selecting studies for inclusion in systematic reviews in general [28] and in meta-ethnographies in particular [7, 9, 16, 22] have been developed. There is also recent guidance on how to select a particular qualitative synthesis approach to suit a research aim [23, 24] and on the quality appraisal of studies for inclusion in a meta-ethnography [7, 42]. Detailed worked examples [7, 9, 16, 22, 42] of applying meta-ethnography to the conduct of health-related reviews have been published in the 2000s. However, reporting of health-related meta-ethnographies is often poor [15, 18] and few recent meta-ethnographies have cited the above worked examples [18].

Well-conducted, well-reported meta-ethnographies can provide valuable evidence to improve patient experiences, public health, and health services for any health condition, issue or service. To realise the high potential value of meta-ethnography requires high quality, transparent reporting that clearly conveys the methodology, analysis and findings. Current poor reporting [15, 18] is a barrier to meta-ethnography use as users cannot assess its quality and trustworthiness, however, bespoke reporting guidelines can raise reporting quality [29].

To be effective, a reporting guideline must be used. For this to happen, the intended users of the reporting guideline must be aware of the existence, and must trust in the quality, of the guideline and how it was developed. To engender trust in the guideline, it will be created through a rigorous study that follows good practice in reporting guideline development. The content of the guideline will be the culmination of learning from comprehensive literature reviews and debate with and the consensus of academic experts and other stakeholders. The project team will ensure that an audit trail of decisions made at each stage in the study is documented so that the decision-making process can be scrutinised. To raise awareness of the existence of the guideline we have registered our intent to produce these, and will register the final guideline, with the EQUATOR (Enhancing the QUAlity and Transparency Of health Research) international network and comprehensive database of reporting guidelines (http://www.equator-network.org/). The project has also been awarded DECIPHer (Development and Evaluation of Complex Interventions for Public Health Improvement) affiliated status and the team will report project performance and impact to them annually. The methodological systematic review to be conducted in Stage 1 has been registered with the PROSPERO international database (http://www.crd.york.ac.uk/PROSPERO/). Furthermore, we will negotiate with and ask editors of journals that publish meta-ethnographies to promote the guideline by making a clear statement of how the journal expects authors to use the guideline, what level of adherence is required, to consider asking authors to state how their article meets the guideline items and by asking peer-reviewers to use them as part of their review. Journal editors will be asked to notify us when they endorse the reporting guideline to help us to document and track all endorsements. The project team will continue to gather user feedback on use of the guideline to inform its future refinement.

The consensus study will start in May 2016 with an online workshop followed by the eDelphi studies in August 2016 and an article describing the guidelines will be submitted by May 2017. We have established an online discussion forum for anyone with an interest in meta-ethnography which can be found at: www.jiscmail.ac.uk/META-ETHNOGRAPHY. The project website is available at http://www.stir.ac.uk/emerge/.
